# T-shaped dysmorphic uterus: discrepancies between current 3D-ultrasound diagnostic criteria

**DOI:** 10.1007/s00404-025-07986-9

**Published:** 2025-03-21

**Authors:** Giulia Monaco, Elvira Nocita, Aikaterini Selntigia, Consuelo Russo, Daniele Farsetti, Sabrina Reppuccia, Carlo De Angelis, Brunella Zizolfi, Attilio Di Spiezio Sardo, Caterina Exacoustos

**Affiliations:** 1 Department of Surgical Sciences Obstetrics and Gynecological Clinic, University of Rome Tor Vergata, Viale Oxford, 81, 00133 Rome, Italy; 2 Department of Women, Children, and Public Health Sciences, Fondazione Policlinico Universitario A. Gemelli IRCCS, Rome, Italy; 3 Department of Public Health, University of Naples Federico II, Naples, Italy; 4 Department of Maternal and Child Health and Urological Sciences, University of Rome Sapienza, Rome, Italy

**Keywords:** Uterine congenital anomalies, T-shaped uterus, 3D transvaginal ultrasound

## Abstract

**Purpose:**

Review and compare current classifications for diagnosing T-shaped uterus using three-dimensional transvaginal ultrasound (3D-TVS) measurements, identifying measurements that best correlate with the diagnosis.

**Methods:**

This retrospective cohort study analyzed diagnostic measurements in patients with T-shaped uterus who underwent 3D-TVS at the University of Rome ‘Tor Vergata’ from 2016 to 2022. Of 7588 patients, four sonographers re-evaluated 3D-TVS images of 72 initially diagnosed cases. Uterine morphology was assessed in the coronal plane by measuring fundal cavity width (R0), corpus-isthmic cavity width (Wi), lateral indentation angle (AI), lateral bulging (LB), T-angle (AT), fundal/isthmic cavity width ratio (R0/Wi), and the length of intracavitary line parallel to interostial line (R10). All cases were confirmed by hysteroscopy, excluding ambiguous findings.

**Results:**

Of 72 initially evaluated patients, 50 met the inclusion criteria for final analysis. These patients had consistent 3D-TVS diagnoses from four sonographers and hysteroscopic confirmation from two experts. The combination of three CUME criteria (AT ≤ 40°, AI ≤ 130°, LB ≥ 7 mm) identified only 8% of T-shaped uteri. Notably, 30 patients (60%) had an R10 measurement of ≤ 10 mm. In addition, 31 uteri (62%) met all three criteria: LB ≥ 5 mm, AI ≤ 140°, and R0/Wi ≥ 5. Overall, 48 uteri (96%) satisfied at least two criteria. The study concluded that LB, R10, and R0/Wi are independent predictors of T-shaped uterus.

**Conclusions:**

Significant discrepancies exist among current classifications for diagnosing T-shaped uterus. This study identified LB, R10, and R0/Wi as key parameters for accurate diagnosis. These measurements provide a precise and objective approach, aiding in the evaluation of the anomaly's impact on reproductive outcomes and the benefits of hysteroscopic treatment.

**Supplementary Information:**

The online version contains supplementary material available at 10.1007/s00404-025-07986-9.

## What does this study add to the clinical work

**Table:** 

This work proposes new combination of 3D ultrasound diagnostic criteria that can allow a correct and reproducible definition of the T-shaped uterus.	

## Introduction

The T-shaped uterine malformation is a rare Müllerian anomaly, and the estimation of its exact prevalence is unknown. An accurate diagnosis of T-shaped uterus still represents a challenge due to the non-systematic use of diagnostic methods with varying accuracy and the impact of the hysteroscopic treatment remains questionable [1–13].

The more recent classifications (AFS 1988, ESHRE/ESGE 2016, ASRM 2021) [14–18] for uterine congenital anomalies did not include or describe accurately diagnostic criteria for this uterine malformation. Only the ESHRE/ESGE 2013 [19] proposed to include the T-shaped uterus in Class U1a, a sub-category of the dysmorphic uteri.

Since the best diagnostic method for uterine congenital abnormalities was identified in the 3D coronal view obtained by TVS, different diagnostic measurements have been suggested to correctly identify T-shaped uteri. In 2015, Exacoustos et al. [20] proposed at least two out of three measurements for the diagnosis of T-shaped uterus (lateral indentation angle (AI) ≤ 140°, lateral bulging(LB) ≥ 5 mm and fundal/isthmic cavity width ratio(R0/Wi ≥ 5). In 2020, Ludwin et al. [21] proposed another diagnostic 3D ultrasound model based on the presence of all three criteria: AI ≤ 130°, LB ≥ 7 mm and T angle(AT) ≤ 40°. Furthermore, Pacheco et al. [22] proposed the so called “Rule of 10” to diagnose T-shaped uterus where the thickness of uterine cavity was ≤ 10 mm from a linear measurement of 10 mm from the interostial line.

Based on the discrepancies between the current classification, the aim of this study is to review and compare these three current diagnostic models for the diagnosis of T-shaped uterus and to identify the best 3D-TVS measurements and models that best correlates to its diagnosis.

## Methods

### Study design

**Fig. 1:**
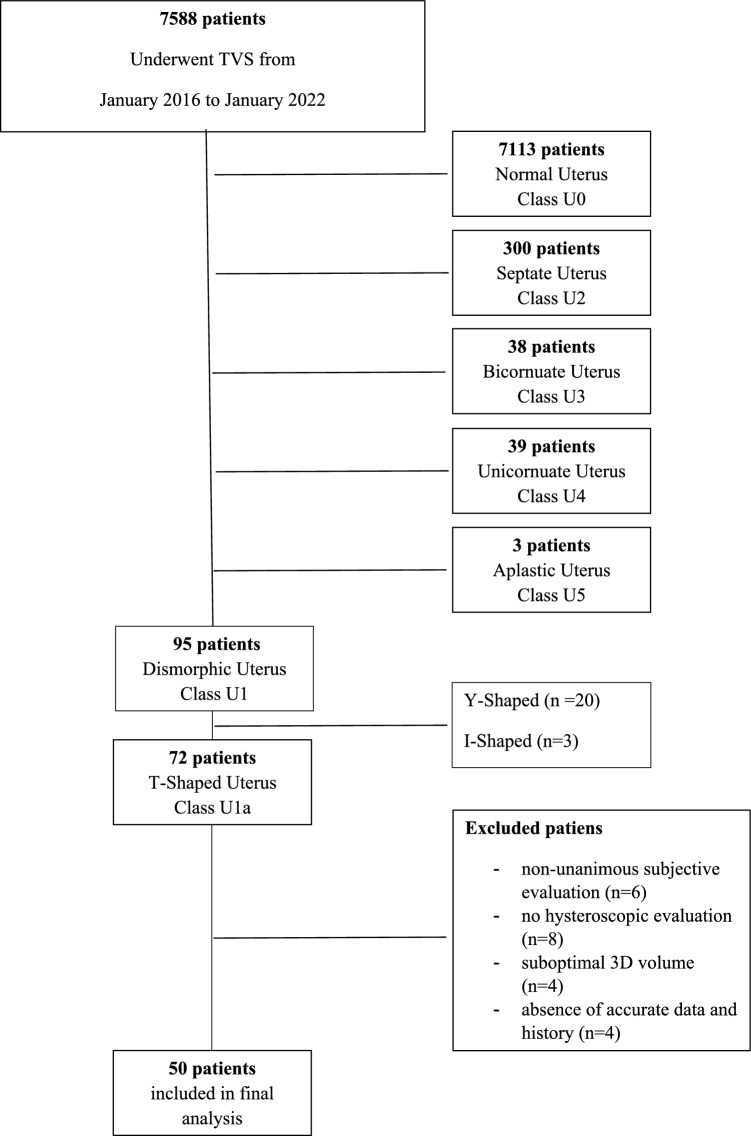
Study design

In this retrospective study, we reassessed offline uterine volumes of women referred to the Gynecological Ultrasound Unit at the University of Rome ‘Tor Vergata’ from 2016 to 2022. Only patients with a confirmed T-shaped uterus, verified by four experienced sonographers and hysteroscopy, were included. All patients underwent 2D, 3D, and Power Doppler TVS examinations during the secretory phase of the menstrual cycle. To assess measurement concordance, only those with a subjective 3D-TVS diagnosis and hysteroscopic confirmation were included. Exclusion criteria included the absence of hysteroscopic evaluation, suboptimal 3D images, uncertain T-shaped cavity, fundal indentation > 3 mm, incomplete reproductive history, ongoing pregnancy, menopause, malignancy, benign uterine pathologies, previous surgeries, and hormonal treatments (Fig. 1).

**Fig. 2:**
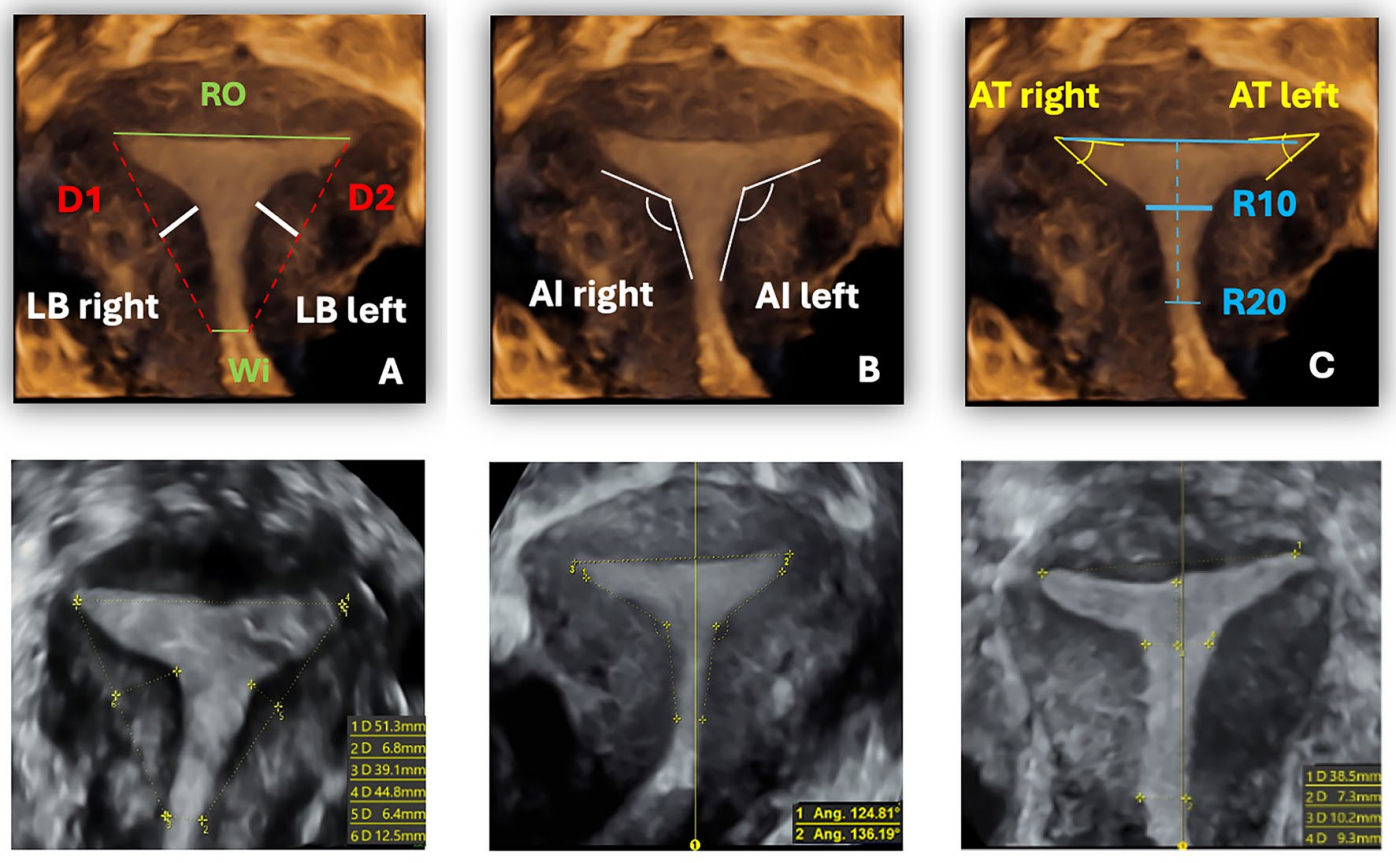
Uterine cavity measurements on transvaginal ultrasound 3D coronal view in T-shaped uteri according to the classifications currently in use. **a**—R0: fundal cavity width, the distance between the two internal tubal ostia.—Wi: corpus isthmic level cavity width, width of uterine cavity at the isthmic level or internal uterine orifice.—D1 right; D2 left: Distance between the tubal ostium and uterine isthmus/internal orifice.—LB right; LB left: lateral bulging, distance between D (right and left) and ipsilateral indentation angle AI. **b **—AI right; AI left: lateral indentation angle, the lateral angle between the corpus-isthmic cavity and the two fundal endometrial layers. **c**-AT right; AT left: T angle, the lateral angle between most lateral point of uterine cavity, contralateral most lateral point of uterine cavity and apex of ipsilateral lateral indentation.—R10: length of the intracavitary line parallel to the interostial line at 10 mm from it.—R20: length of the intracavitary line parallel to the interostial line at 20 mm from it

The included T-shaped uteri were evaluated according several measurements defined in the three current different diagnostic models: Exacoustos et al. 2015, Luwin et al. 2020 and Alonso-Pacheco 2021 [20–22]. The stored volume of the T-shaped uteri confirmed by subjective evaluation offline and by hysteroscopy were analyzed on coronal section using the several measurements proposed by all different diagnostic classifications currently in use (Fig. 2).

### Clinical history and symptoms

Patient data was recorded in FileMaker Pro® 9.0. The collected information included date of birth, age at ultrasound, BMI, age at menarche, parity, menstrual cycle details, last menstrual period, previous surgeries, endocrinological conditions, autoimmune diseases, childhood illnesses, and family history of metabolic and oncological diseases. Patients attempting to conceive provided reproductive history, including previous pregnancies (spontaneous or assisted), delivery complications, and delivery type. Reproductive complications were defined as follows: preterm birth before 37 weeks of gestation [23], miscarriage as loss of pregnancy within the first 23 weeks, recurrent miscarriage as two or more losses within that timeframe, infertility as trying to conceive for one year or longer without male infertility factors [24], and ectopic pregnancy as any implantation outside the endometrial cavity [25].

### Ultrasound examination

Ultrasound exams were performed using a Voluson E6 or E8 (GE Healthcare) with a transvaginal probe and standardized settings. Initially, a conventional 2D ultrasound assessed the pelvis, including the uterus, endometrium, and adnexa, documenting any abnormalities. This was followed by 3D-volume acquisition, capturing two to four static grayscale volumes from transverse and/or sagittal planes to optimize the coronal view. Multiple acquisitions were made to minimize the effects of uterine contractions. Datasets were stored on DVDs for offline analysis. Uterine architecture was analyzed on a standardized coronal plane, using the interstitial portions of the fallopian tubes and internal tubal ostia as reference points, which enhanced visualization of the cervical isthmus and internal cervical orifice.

### Study group

Only patients with a T-shaped uterus, confirmed by offline subjective evaluation from four sonographers and subsequent hysteroscopic diagnosis, were included for further analysis.

Specific measurements were performed offline on the T-shaped cavity using the 3D coronal view (Fig. 2):

- fundal cavity width (R0) (distance between the two internal tubal ostia) (Fig. 2a);
- width of uterine cavity at the isthmic level or internal uterine orifice (Wi- corpus isthmic level cavity width) (Fig. 2a);
- fundal/isthmic cavity width ratio (R0/Wi) (Fig. 2a);
- distance between the tubal ostium and uterine isthmus/internal orifice (D1 right; D2 left) (Fig. 2a);
- distance between D1 and indentation angle AI (LB) (Fig. 2a);
- lateral angle between the corpus-isthmic cavity and the two fundal endometrial layers (indentation angle: AI right; AI left) (Fig. 2b);
- lateral angle between most lateral point of uterine cavity, contralateral most lateral point of uterine cavity and apex of ipsilateral lateral indentation (T angle AT) (Fig. 2c).
- length of the intracavitary line parallel to the interostial line at 10 mm from it (R10) (Fig. 2c).

All these measurements are those used alone or in combination by the three current models for the identification of T-shaped proposed by Exacoustos et al. 2015, Ludwin et al. (CUME) 2020 and Alonso-Pacheco 2021 [20–22].

Hysteroscopy was performed on all patients by two experts (CDA and ADS) during days 6 − 10 of the menstrual cycle. A T-shaped uterus was diagnosed if there was an increased distance between the tubal ostia, absence of fundal indentation, a narrow isthmic cavity, or an elongated cervical canal, following a 2/3 uterine corpus to 1/3 cervix ratio. Interostial distance and transverse diameter at the isthmus were measured using 5 Fr grasping forceps (6 mm) as a reference [12].

### Statistical analysis

Statistical analyses were performed using MedCalc software. Qualitative variables were presented as counts (n) and percentages (%). Differences in quantitative variables were assessed with the two-tailed Student’s t test, while the Chi-squared test compared proportions. A p value < 0.01 was deemed significant.

Diagnostic accuracy of the Exacoustos, CUME, and Alonso-Pacheco models [20–22], along with their cutoffs, was evaluated using ROC analysis. Areas Under the Curve (AUC) were compared. Univariate and multivariate logistic regression analyses identified the best sonographic predictors for a T-shaped uterus.

## Results

Among the 7588 patients seen between 2016 and 2022, 7113 had a normal uterine cavity (Class U0 according to the ESHRE/ESGE classification, 300 had septate uteri (Class U2), 38 had bicornuate uteri (Class U3), 39 had unicornuate uteri (Class U4), 3 had aplastic uteri (Class U5), and 95 had dysmorphic uteri (Class U1) [19].

The dysmorphic group included 20 Y-shaped, 3 I-shaped, and 72 T-shaped uteri. Of the 72 patients initially diagnosed with a T-shaped uterus, 50 met the inclusion criteria and were enrolled in this study. Six patients were excluded due to non-unanimous evaluations among the sonographers, eight were excluded because hysteroscopic evaluation was not performed, four due to suboptimal 3D volume, and four due to incomplete data and history (Fig. 1).

Table 1 outlines the characteristics, ultrasound indications, and reproductive history of 50 patients. Indications for TVS included: 23 patients (46%) for infertility and recurrent pregnancy loss, 15 (30%) for pelvic pain/endometriosis, and 8(16%) for suspected congenital uterine anomalies. Of the patients, 31 (62%) were infertile (21 with primary and 10 with secondary infertility), 5 (10%) had at least 1 miscarriage, and 2 (4%) had recurrent miscarriages.

**Table 1:** Characteristics, indications to ultrasound evaluation and reproductive history of the 50 patients with T-shaped uterus at 3D-TVS and hysteroscopy included in this study population at the time of the diagnosis

Patients’ characteristics at diagnosis (*n* = 50)	Mean ± SD *n* (%)
Age (years)	36.8 ± 5.8
Bmi	22.6 ± 6.2
Menarche (years)	12.1 ± 1.4
Gravidity	0.7 ± 0.9
*Parity*	± 0.3
Nulliparity	45 (90%)
Multiparity	5 (10%)
*Indication to TVS*	
Pelvic pain	10 (20%)
Suspected endometriosis	5 (10%)
Irregular menstrual cycle	4 (8%)
Suspected uterine anomalies	8 (16%)
Infertility	21 (42%)
Recurrent pregnancy loss	2 (4%)
*Reproductive problems*	
**Miscarriages**	**5 (10%)**
One miscarriage	3 (6%)
≥ 2 miscarriages	2 (4%)
**Infertility**	**31 (62%)**
Primary infertility	21 (42%)
Secondary infertility	10 (20%)

Bold values represent subcategories within the table

*TVS* transvaginal sonography, *SD* standard deviation

Table 2 summarizes the performance of each individual measurement and the combination of multiple criteria. Among the three criteria proposed by CUME [21], the AI ≤ 130° demonstrated the highest concordance rate (64%) for diagnosing T-shaped uterus, with an AUC of 0.820 (0.749–0.878). In contrast, the T-angle (AT) ≤ 40° showed the lowest concordance, with an AUC of 0.570 (0.487–0.650). Pairwise comparison of ROC curves confirmed that the T-angle ≤ 40° had the poorest diagnostic performance. According to the predictive model proposed by Ludwin [21], only 4 uteri (8%) in our cohort met all three criteria (LB ≥ 7 mm, AI ≤ 130°, AT ≤ 40°), yielding an AUC of 0.540 (0.457–0.622) (Table 2). The presence of at least two of the three CUME criteria identified 42% of T-shaped uteri, with an AUC of 0.710 (0.630–0.781).

**Table 2:** Comparison of different measurements and diagnostic criteria of current classifications system in the detection of T-shaped uterus. Finally, the new proposal of this study and the association of more criteria to diagnose T-shaped is reported (AUC area under the curve, CI confidence of interval)

Current classifications	T- shaped (*n* 50)	Concordance rate	AUC	95% CI
*Ludwin 2020 – CUME*				
Lateral indentation angle (AI) ≤ 130°	32	64%	0.820	0.749–0.878
T-angle (AT) ≤ 40°	7	14%	0.570	0.487–0.650
Lateral bulging (LB) ≥ 7 mm	24	48%	0.740	0.662 −0.808
All of three	4	8%	0.540	0.457–0.622
At least two of three	21	42%	0.710	0.630–0.781
At least one of three	38	76%	0.880	0.817–0.927
*Alonso Pacheco 2021 – RULE OF 10*				
R10 ≤ 10 mm	30	60%	0.795	0.721–0.856
*Exacoustos 2015*				
Lateral indentation angle (AI) ≤ 140°	44	88%	0.930	0.877–0.965
Lateral bulging (LB) ≥ 5 mm	48	96%	0.970	0.928–0.991
RO/Wi ≥ 5	37	74%	0.840	0.771–0.895
All of three	31	62%	0.810	0.738–0.869
At least two of three	48	96%	0.980	0.943–0.996
At least one of three	50	100%	0.950	0.902–0.979
*This Study*				
R10 ≤ 10 mm	30	60%	0.795	0.721–0.856
Lateral bulging (LB) ≥ 5 mm	48	96%	0.970	0.928–0.991
RO/Wi ≥ 5	37	74%	0.840	0.771–0.895
All of three	17	34%	0.670	0.589–0.745
At least two of three	48	96%	0.980	0.943–0.996
At least one of three	50	100%	0.955	0.908–0.982

In line with the “rule of 10” proposed by Alonso-Pacheco [22], R10 ≤ 10 mm was observed in 30 uteri (60%), showing a diagnostic test accuracy with an AUC of 0.795 (0.721–0.856) (Table 2).

Considering the criteria proposed by Exacoustos [20] (LB ≥ 5 mm, AI ≤ 140°, R0/Wi ≥ 5), 31 uteri (62%) met all three criteria, and 48 uteri (96%) fulfilled at least two of the three criteria. Among the individual criteria, AI ≤ 140° demonstrated an AUC of 0.930 (0.877–0.965). Pairwise comparison of ROC curves showed that AI ≤ 140° had better diagnostic performance compared to AI ≤ 130° (*p* < 0.001). The LB ≥ 5 mm showed the highest accuracy, with an AUC of 0.970 (0.928–0.991). Pairwise comparison confirmed that LB ≥ 5 mm had superior diagnostic performance compared to LB ≥ 7 mm (*p* < 0.001). The diagnostic model based on all three Exacoustos criteria had an AUC of 0.810 (0.738–0.869). However, the presence of at least two criteria provided the best accuracy for identifying a T-shaped uterus, with an AUC of 0.980 (0.943–0.996) (Table 2).

**Fig. 3:**
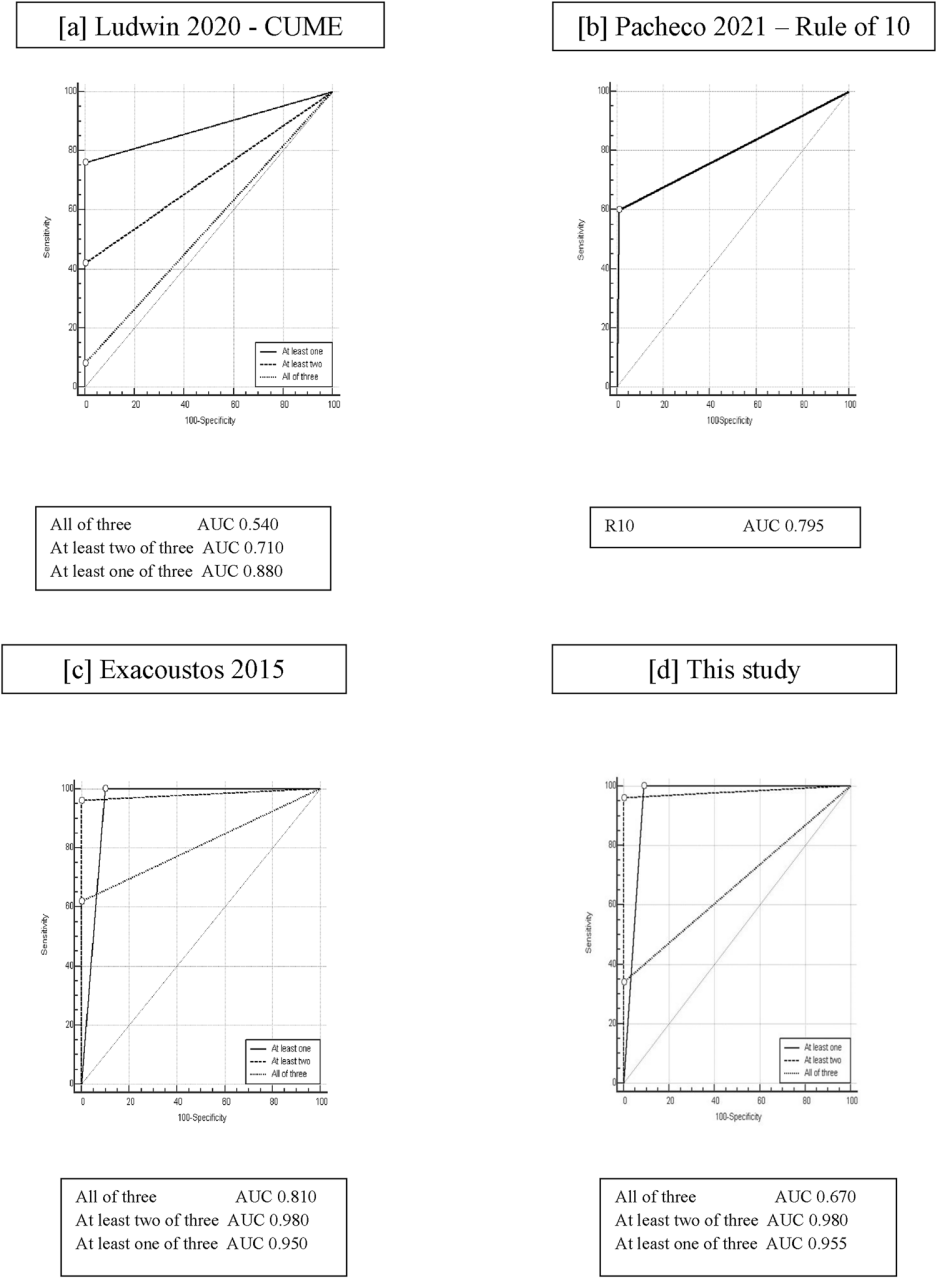
Comparison between ROC curves of the current classifications and the new proposal. **a** Ludwin 2020 – CUME [21]. In the predictive model proposed all the three criteria together (LB ≥ 7 mm, AI ≤ 130°, AT ≤ 40°) showed an AUC of 0.540 (0.457–0.622). The presence of at least two of the three criteria identified an AUC of 0.710 (0.630–0.781), and at least one of the three criteria showed an AUC = 0 .880 (0.817–0.927). **b** Alonso Pacheco 2021 – Rule of 10 [22] The diagnostic test accuracy showed an AUC of 0.795 (0.721–0856). **c** Exacoustos 2015 [20]. The diagnostic model with all three criteria showed an AUC 0.810 (0.738–0.869). The presence of at least two criteria showed the best accuracy in identification of T-shaped uterus with AUC of 0.980 (0.943–0.996), while the presence of at least one criterion identified T-shaped uterus had an AUC of 0.950 (0.902–0.979) **d** This study. The presence of the three criteria together identified an AUC of 0.670 (0.589–0.745). The presence of at least two criteria identified an AUC of 0.980 (0.943–0.996). The presence of at least one criterion identified 100% of the uteri with an AUC of 0.955 (0.908–0.982)

**Fig. 4:**
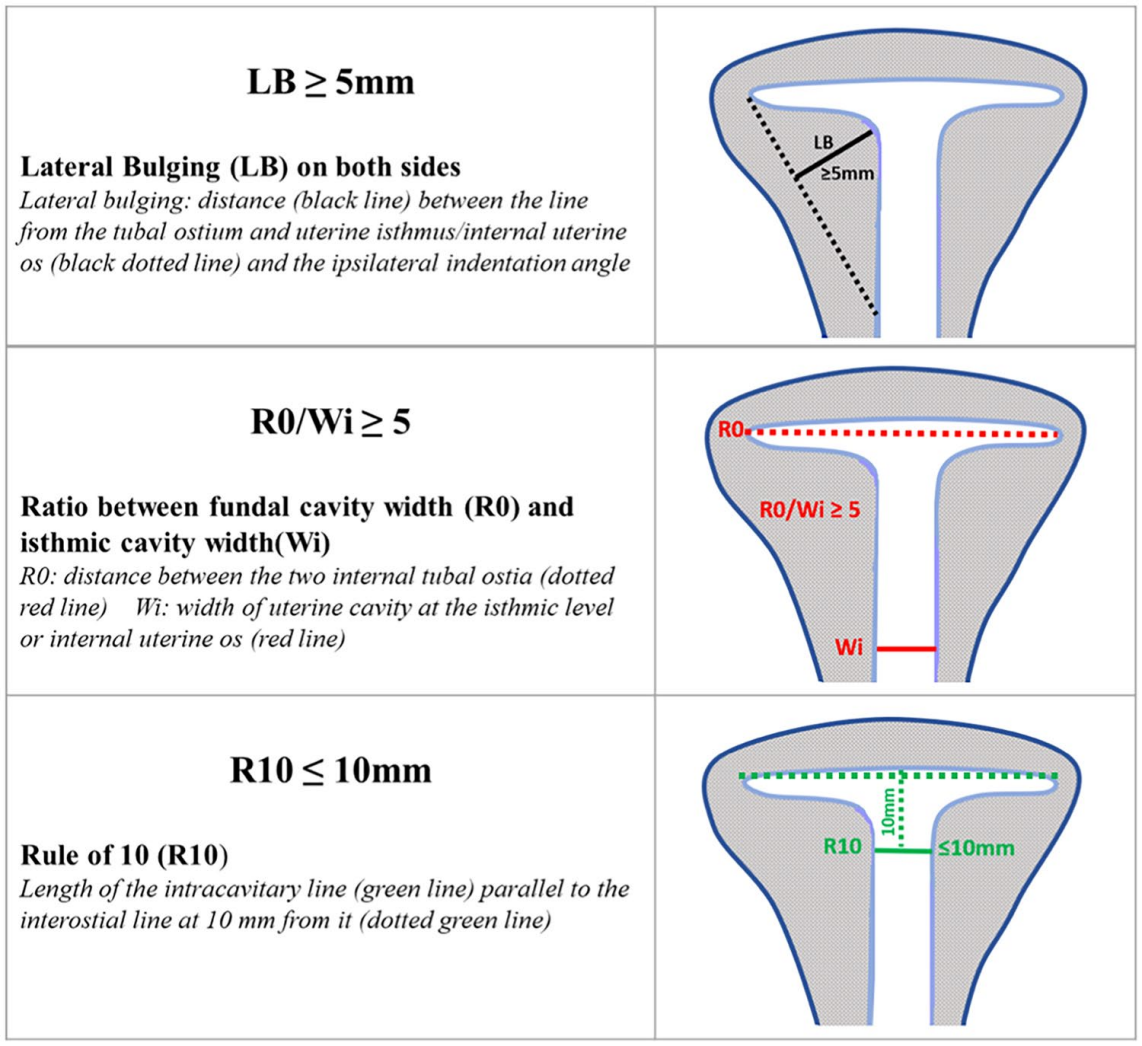
Measurement cut-offs for the diagnosis of T-shaped uterus on 3D-TVS coronal section: at least two of these measurement cut-offs should be present (better all three)

Finally, through multivariate regression analysis (Fig. 3), we identified three sonographic features as independent predictors for a T-shaped uterus: LB, R10, and R0/Wi. A new diagnostic model was created using these variables, with cutoffs selected based on the highest diagnostic accuracy among those previously validated: LB ≥ 5 mm, R10 ≤ 10 mm, and R0/Wi ≥ 5. The combination of all three criteria identified 17 uteri (34%), with an AUC of 0.670 (0.589–0.745). However, the presence of at least two of these criteria identified 96% of the uteri, with an AUC of 0.980 (0.943–0.996), in particular, LB + R0/Wi was found in 36 uteri (72%), LB + R10 in 28 uteri (56%), and R0/Wi + R10 in 18 uteri (36%) (Table 2). Therefore, the most effective diagnostic model for identifying a T-shaped uterus is the presence of at least two of the following criteria: LB ≥ 5 mm, R10 ≤ 10 mm, and R0/Wi ≥ 5 (Fig. 4).

## Discussion

The diagnosis of a T-shaped uterus is often subjective and problematic due to the lack of precise criteria across different classification systems. The need to establish clear and reproducible diagnostic criteria for identifying a T-shaped uterus arises from the necessity to understand its true impact on fertility. Several studies [1–3] have reported poor reproductive outcomes in patients with a T-shaped uterus, although the pathophysiology behind these adverse outcomes remains unclear. Recently, an increasing number of studies have evaluated the impact of hysteroscopic metroplasty in patients with a T-shaped uterus. Some of these studies have described improvements in reproductive outcomes following the restoration of normal uterine cavity anatomy [3–12]. However, there is an open debate on this of treatment often due to the lack of preoperative accurate diagnosis. As indicated in the article of Carton et al. [13], spontaneous pregnancy can occur after hysteroscopic metroplasty but CUME ultrasound criteria [21] do not appear to be predictive of post-surgery outcomes.

In this study, we compared ultrasound diagnostic criteria for T-shaped uterus among a cohort classified by four operators, seeking the best cut-offs to identify this morphology. To ensure accurate evaluation, we included only cases with hysteroscopic confirmation of T-shaped uteri based on specific criteria, supporting the initial subjective 3D-TVS diagnoses. However, we acknowledge that 3D-TVS is considered the gold standard for diagnosing congenital uterine malformations. The 3D-volume acquisition and subsequent coronal section reconstruction do not actually require highly specialized expertise. However, the correct classification requires a clear understanding of the types of uterine anomalies and how to obtain an objective diagnosis of the different congenital anomaly classes.

To accurately evaluate Müllerian anomalies, both the uterine cavity and external profile should be assessed. MRI can be an alternative to 3D-TVS coronal sections, offering the same views and measurements for diagnosing and classifying these anomalies. For a T-shaped uterus, the tubular isthmic cavity can be seen through hysterosalpingography or hysteroscopy, but it is also important to evaluate the thickness of the lateral myometrium and the external shape. Specifically, LB, rather than the angle, is crucial for guiding hysteroscopic surgery.

Although rare, we identified 50 typical T-shaped uteri in our cohort, evaluated subjectively by four sonographers using 3D-TVS and one of two expert hysteroscopists. Ambiguous T-shaped uteri and those with fundal indentation (Y-shaped) were excluded. After selection, we applied on the coronal plane measurements from three different authors **(**Fig. 2**)**.

We found that LB ≥ 5 mm, R0/Wi ≥ 5, and R10 ≤ 10 mm are the most accurate measurements associated with T-shaped uterine morphology. Uterine side wall thickness, the tubular isthmic aspect, and the flat fundal cavity are key parameters for dysmorphic T-shaped uteri according to all classification systems (Fig. 4). The increased side wall thickness, which gives the cavity a tubular shape, is reflected in the LB and AI measurements. While both are comparable, we chose to use LB because it is easier to measure.

Consistent with Exacoustos et al. [20], we found that LB ≥ 5 mm correlates better with T-shaped uteri than LB ≥ 7 mm, which is why we included this criterion for describing T-shaped morphology. To objectively assess the typical tubular cavity, the “Rule of 10” [22] offers a simple and reproducible method. In our study population, applying the R10 identified T-shaped uteri in 60% of cases.

Considering the tubular isthmic cavity and the flat, wide fundal cavity as specific features of T-shaped uteri, the R0/Wi and R10 provide objective measurements to differentiate between normal and T-shaped uteri. Evaluating all parameters and cut-offs from three different classifications, we found that the T-angle proposed by CUME had the lowest diagnostic accuracy, present in only 4 out of 50 uteri [21]. The combination of CUME parameters correlated with our selected T-shaped uteri in only 8% of cases and in 42% when using two criteria (doubtful T-shaped).

In contrast, using only the R10 from Pacheco [22] identified 60% of T-shaped uteri. Exacoustos et al. [20] classification score, incorporating three criteria, missed 38% of T-shaped cases.

These discrepancies across the three classifications highlight the absence of clear, objective diagnostic criteria, making the accurate diagnosis and differential diagnosis of T-shaped uterus more challenging. Through multivariate analysis, we aimed to identify 3D-TVS measurements with optimized cut-offs for the best diagnostic correlation. We found that the combination of at least two out of three criteria (LB ≥ 5 mm, R0/Wi ≥ 5, and R10 ≤ 10 mm) provided the highest diagnostic precision, with the association of LB ≥ 5 mm and R0/Wi ≥ 5 being the most effective (72%).

The main limitation of this study is its retrospective design, which may result in missing data. In addition, the lack of comparisons with other uterine anomalies and normal cavities, as well as the absence of prevalence data for T-shaped uteri in the general population, are notable limitations.

We therefore performed a statistical evaluation on the 50 identified typical T-shaped uteri in our cohort. However, to apply a correct sample-size calculation, further prospective studies with a larger number of cases will be needed to increase the accuracy of the measurements. However, the primary goal of this study was to identify clear and objective 3D-TVS parameters that correlate with T-shaped uteri, enabling their prospective application in the general population. This approach could help assess the true prevalence of this anomaly, especially in subgroups like infertile patients or those with recurrent pregnancy loss.

Defining T-shaped uteri through objective 3D-TVS measurements could clarify the impact of surgical treatments, as current studies show varying outcomes due to different presurgical methods. The study’s strengths include diagnoses by four blinded expert ultrasound examiners, hysteroscopic confirmation by skilled endoscopists, a large sample size despite the rarity of the malformation, and a robust multivariate analysis. These findings have significant clinical implications, improving ultrasound diagnostics and reproductive counseling for T-shaped uterine malformations. This study sets the stage for future evaluations of fertility outcomes, though further prospective studies are needed for validation.

## Conclusion

The 3D coronal section measurements identified in this study for diagnosing a T-shaped uterus are:

- LB ≥ 5 mm;
- R0/Wi ≥ 5;
- R10 ≤ 10 mm.

Meeting at least two of these three criteria enhances the diagnostic performance of ultrasound for detecting a T-shaped uterus (Fig. 4).

The use of clearly defined and consistent diagnostic criteria for T-shaped uterus allows for accurate diagnosis of this uterine dysmorphism. A correct and reproducible diagnosis based on objective measurements, rather than solely on subjective impressions, allows for the comparison of data and results. It also ensures that surgical treatment is considered only for patients who actually have a T-shaped uterus and require hysteroscopic metroplasty due to their symptoms. In fact, a T-shaped uterus is a rare condition and is often overestimated due to an unclear diagnosis. As a result, clinical implications and treatments cannot be adequately compared.

Our study, which defines objective parameters for the correct diagnosis of a T-shaped uterus, lays the groundwork for future evaluations of fertility and reproductive outcomes, as well as the impact of surgery in patients with T-shaped uterus.

## Supplementary Information

Below is the link to the electronic supplementary material.

Supplementary file1 (PDF 19 KB)

## Data Availability

No datasets were generated or analysed during the current study.
